# Development and In Vitro Evaluation of Gefitinib–Salicylic Acid Nanocrystals for Improved Bioavailability

**DOI:** 10.3390/pharmaceutics18050572

**Published:** 2026-05-04

**Authors:** Ling Chen, Jie-Feng Chen, Rong Wang, Tian-Ran Yang, Hao Meng, Xin-Xin Zhu, Hai-Li Wu, Jie-Jie Lai, Wei-Wei Chen, Ning Lin, Qing Chen

**Affiliations:** 1 College of Pharmacy, Institute of Traditional Chinese and Zhuang-Yao Ethnic Medicine, Guangxi University of Chinese Medicine, Nanning 530200, China; chenling2023@stu.gxtcmu.edu.cn (L.C.); chenjiefeng2024@stu.gxtcmu.edu.cn (J.-F.C.); chenqing@gxtcmu.edu.cn (Q.C.); 2 Guangxi Innovation Center of Zhuang Yao Medicine, Guangxi University of Chinese Medicine, Nanning 530200, China

**Keywords:** gefitinib, antisolvent precipitation, nanocrystals, oral bioavailability

## Abstract

**Background**: Non-small cell lung cancer (NSCLC), a malignant tumor with high global incidence and mortality rates, urgently requires more effective targeted drug delivery systems for its treatment. As an EGFR tyrosine kinase inhibitor, gefitinib has its clinical efficacy limited by poor solubility and low bioavailability. This study aimed to develop a gefitinib–salicylic acid salt (Gef-Sa) and its nano-formulation (Gef-Sa-NPs) via a combined strategy of crystal engineering and nanotechnology to improve its pharmaceutical properties. **Methods**: Gef-Sa was prepared using a suspension method, and its salt formation and thermal stability were predicted by the ΔpKa rule and confirmed by various solid-state characterization techniques, including single crystal/powder X-ray diffraction, thermal analysis, and infrared spectroscopy. Gef-Sa-NPs were prepared via an ultrasound-assisted anti-solvent precipitation method. Their performance was evaluated through in vitro dissolution tests, pharmacokinetic studies, and in vitro antitumor experiments. **Results**: Gef-Sa-NPs with a particle size of 31 nm (PDI = 0.15) were successfully prepared. In vitro dissolution tests demonstrated that the nano-formulation exhibited a significantly higher dissolution rate in pH 1.2, pH 4.5, pH 6.8 and pure water when compared with the raw drug (*p* < 0.01). Pharmacokinetic studies revealed that Gef-Sa and Gef-Sa-NPs increased the oral bioavailability in rats to 1.5-fold and 1.9-fold that of the raw drug, respectively. In vitro antitumor experiments confirmed that the Gef-Sa-NPs increased the inhibition rate against A549 cells compared with the Gef. **Conclusions**: This study innovatively combines salt formation and nanonization technologies to systematically address the key issue of the poor solubility of Gef. The resulting nano-formulation demonstrates excellent dissolution characteristics, pharmacokinetic behavior, and antitumor efficacy. This strategy not only provides a novel drug delivery system with translational potential for NSCLC treatment but also offers a paradigm for the formulation design of poorly soluble drugs. Subsequent research will focus on scaling up production and evaluating pre-clinical safety.

## 1. Introduction

Lung cancer is a leading cause of cancer-related mortality globally, with non-small cell lung cancer (NSCLC) accounting for approximately 85% of cases [1,2]. While EGFR tyrosine kinase inhibitors (TKIs) like gefitinib have revolutionized the treatment of advanced NSCLC, their clinical efficacy is often hampered by poor aqueous solubility and low oral bioavailability [3]. Using first-generation gefitinib (Gef) as an example, we find that its limited solubility and intestinal absorption result in suboptimal systemic exposure, constituting a major bottleneck in its clinical utility [4,5]. Therefore, developing strategies to enhance the solubility and bioavailability of gefitinib is crucial for improving its therapeutic outcomes [6].

In oral drug delivery systems, drug solubility and dissolution rate are widely recognized as critical rate-limiting steps for gastrointestinal absorption [7,8]. It is reported that approximately 40% of marketed drugs and up to 75% of candidates under development suffer from poor aqueous solubility, which severely restricts their systemic absorption and ultimate therapeutic effectiveness [9]. To overcome this pervasive challenge, various pharmaceutical strategies have been developed. Among these, crystal engineering and nanotechnology have attracted considerable attention as two highly promising technological pathways [10,11,12].

In pharmaceutical sciences, crystal engineering provides powerful strategies for modulating the physicochemical properties of active pharmaceutical ingredients (APIs) [13]. Among these, salt formation stands as the most established and prevalent approach [14,15]. It is estimated that approximately 50% of marketed drug molecules are administered as salts, as this strategy can systematically enhance critical properties such as solubility, stability, and processability [16]. Notable examples include nintedanib esylate, which possesses high solubility [17], and vortioxetine hydrobromide, known for its favorable properties in tablet formulation [18]. The selection of an appropriate salt form is often guided by the ΔpKa rule, which serves as a crucial theoretical framework for predicting salt formation and for optimizing the dissolution rate and polymorphic stability of ionizable drugs [19].

Furthermore, nanotechnology is recognized as one of the most promising strategies for improving the dissolution characteristics of poorly soluble drugs [20,21,22]. The core mechanism involves reducing drug particles to the nanoscale (typically 1–1000 nm), significantly increasing their specific surface area, which, according to the Noyes–Whitney equation, markedly enhances dissolution rate and solubility [23]. Among various nano-formulations—and compared with formulation technologies such as lipid-based preparations, solid dispersions and polymeric nanoparticles—nanocrystal technology offers the advantages of higher drug loading, absence of excessive carrier materials, a simpler preparation process, and greater suitability for high-dose antitumor drugs [24]. A prominent example is paliperidone palmitate, a long-acting injectable that leverages nanocrystal technology to form a concentrated repository at the injection site, enabling a sustained drug release over several weeks [25]. Despite the considerable promise of these individual technologies, most existing studies treat them as separate approaches. Systematic research on the combined application of crystal engineering and nanotechnology remains relatively scarce [26,27].

**Figure 1:**
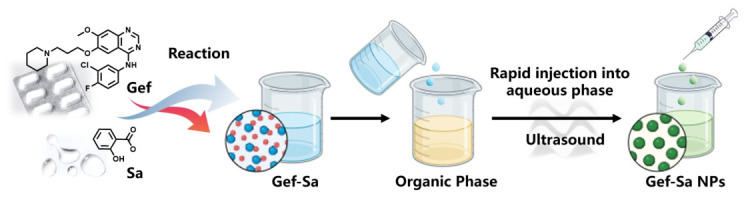
Schematic diagram of Gef-Sa and Gef-Sa-NP preparation.

This study presents an integrated strategy that systematically combines crystal engineering and nanotechnology to enhance the pharmaceutical performance of gefitinib (Figure 1). The approach involved first preparing and characterizing a gefitinib–salicylic acid salt (Gef-Sa), which was subsequently converted into nanoparticles (Gef-Sa-NPs). The resulting nanoformulation was thoroughly evaluated based on its dissolution behavior, pharmacokinetic profile, and antitumor efficacy. This strategy demonstrates a promising route to overcome the current limitations of gefitinib and may contribute to more effective therapeutic options for lung cancer.

## 2. Experimental Details

### 2.1. Materials

Gefitinib (98%), salicylic acid (98%), and lecithin (98%) were purchased from Meryer Chemical Technology Co., Ltd (Shanghai, China). Methanol (HPLC grade) and triethylamine (analytical grade) was purchased from Spectrum China Ltd. (SinBlank) (Shanghai, China). The human non-small cell lung cancer cell line A549 was obtained from the Key Laboratory of Guangxi University of Chinese Medicine (Nanning, China). Fetal bovine serum (FBS) was purchased from Zhejiang Tianhang Biotechnology Co., Ltd (Hangzhou, China). DMEM/F12 medium was purchased from Gibco Corporation (Waltham, MA, USA). Phosphate buffer saline (PBS), penicillin–streptomycin solution, trypsin-EDTA digest (0.25%), and 3-(4,5-Dimethylthiazol-2-yl)- 2,5-diphenyltetrazolium bromide (MTT) were purchased from Solarbio Biotechnology Co., Ltd (Beijing, China). Other chemicals used were of analytical grade.

The animals were purchased from Hunan Slike Jingda Laboratory Animal Co. Ltd (Changsha, China). (certificate no. SCXK (Xiang) 2023-0004). All of the animals were fed and cared for according to the Principles of Laboratory Animal Care and the Guide for the Care and Use of Laboratory Animals. All of the related experiments were conducted under the guidelines from the Animal Ethics Committee approved protocols of Guangxi University of Chinese Medicine (including approval No. DW20231211-063).

### 2.2. Preparation of Gef-Sa

Gef-Sa was prepared by the suspension method. Gefitinib (446.9 mg, 1 mmol) and salicylic acid (276.2 mg, 2 mmol) were accurately weighed into a penicillin vial, and 10 mL of isopropanol was added. The mixture was magnetically stirred at 25 °C for 24 h. After filtration, the product was dried at room temperature to obtain Gef-Sa powder. The purity of Gef-Sa was calculated by the mass balance method as the ratio of the actual obtained product mass to the theoretical yield, and the purity was 96.0%.

### 2.3. Determination of the Dissociation Constants of Gef and Sa

The dissociation constants of Gef and Sa were determined by spectrophotometry [28]. Buffer solutions with different pH values were prepared using hydrochloric acid and sodium hydroxide and kept for later use. Solutions of Gef and Sa at different pH values were prepared, and the absorbance (*A*) of 8–10 different pH buffer solutions was measured at specific wavelengths in the range of 200 to 400 nm. The experimental data were processed using OriginPro 9.0 software and substituted into the formula *pK*_a_ = pH + log[(*A* − *A*_b_)/(*A*_a_ − *A*)] for calculation, where *A*_a_ and *A*_b_ represent the absorbance values of the acid and base, respectively. The average *pK*_a_ value was obtained through linear fitting.

### 2.4. Prescription Process Optimization and Validation of Gef-Sa-NPs

Initially, single-factor screening experiments were conducted to preliminarily identify the key process and formulation parameters affecting the particle size and stability of the developed formulation (Table S6). Subsequently, three critical factors significantly influencing the preparation process of Gef-Sa-NPs (Gef-Sa concentration, organic-to-aqueous phase ratio, and ultrasonication time) were selected. These factors were modeled and optimized with critical quality attributes (size and PDI) as evaluation indicators. Finally, the formulation optimization was completed using Design Expert 13 software to determine the optimal process conditions [29,30].

### 2.5. Preparation of Gef-Sa-NPs

Gef-Sa-NPs were prepared by the anti-solvent precipitation-ultrasonic method using lecithin as the stabilizer. The preparation was carried out according to the optimal process conditions obtained from single-factor screening: 45 mg of Gef-Sa was dissolved in 5 mL of methanol to form the organic phase, which was rapidly injected into 10 mL of lecithin solution (0.1 mg/mL) under vigorous stirring, followed by probe sonication (220 W, 14 min) in an ice bath. The preparation temperature was controlled at 0–4 °C during nanoparticle formation. Finally, the residual methanol was removed by rotary evaporation under reduced pressure to obtain the Gef-Sa-NP suspension.

## 3. Results and Discussion

### 3.1. Measurement of Dissociation Constant (pKa)

**Figure 2:**
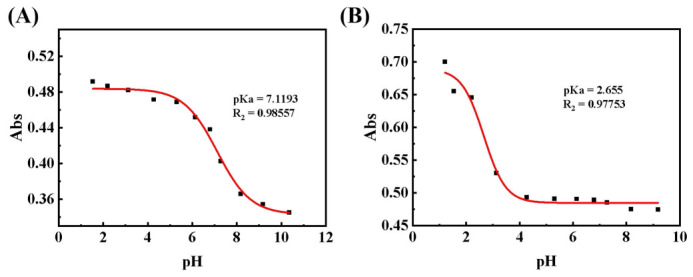
Sigmoidal mathematical model fitting Abs–pH curve of (**A**) gefitinib and (**B**) salicylic acid.

The fundamental distinction between cocrystals and salts lies in whether proton transfer occurs [31]. The ΔpKa rule is commonly used for the preliminary prediction of pharmaceutical multi-component crystals, where ΔpKa = [pKa (protonated base) − pKa (acid)]. Generally, it is accepted that when ΔpKa < 0, proton transfer does not occur, resulting in the formation of a cocrystal; when ΔpKa > 3, proton transfer takes place, leading to salt formation; and when 0 < ΔpKa < 3, either a cocrystal or a salt may form [32]. As shown in Figure 2A,B, the pKa values of Gef and Sa are 7.1193 and 2.655, respectively. The calculated ΔpKa value for the Gef-Sa compound is 4.4643, indicating the successful formation of a salt. Detailed analysis data regarding ΔpKa are provided in Tables S1 and S2.

### 3.2. Optimization of Gef-Sa-NPs

**Figure 3:**
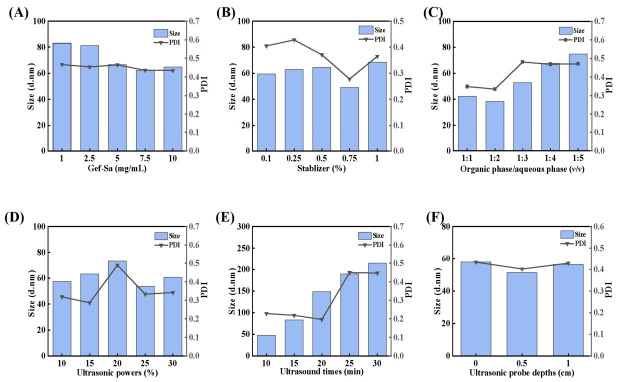
Effect of (**A**) Gef-Sa concentrations, (**B**) stabilizer, (**C**) organic phase/aqueous phase (*v*/*v*) ratios, (**D**) ultrasonic powers, (**E**) ultrasound times, and (**F**) ultrasonic probe depths on size and PDI of Gef-Sa-NPs.

In this study, Gef-Sa-NPs were prepared using an ultrasound-assisted anti-solvent precipitation method [33]. The effects of the various factors shown in Figure 3, including Gef-Sa concentration, stabilizer concentration, and organic-to-aqueous phase ratio, on the properties of the nanoparticles were systematically investigated. Additionally, the particle size and PDI of the NPs were monitored by Malvern particle size potential analyzer. Preliminary experimental results indicated that Gef-Sa concentration, organic-to-aqueous phase ratio, and sonication time had the most significant influence on the nanoparticle properties.

**Figure 4:**
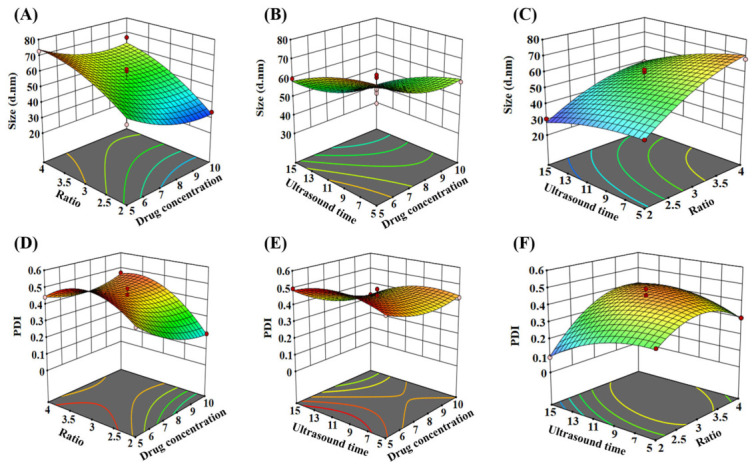
Response surface of (**A**) Gef-Sa concentrations and organic phase/aqueous phase ratios, (**B**) Gef-Sa concentrations and ultrasound time, (**C**) ultrasound time and organic phase/aqueous phase ratios on size of Gef-Sa-NPs. Response surface of (**D**) Gef-Sa concentrations and organic phase/aqueous phase ratios, (**E**) Gef-Sa concentrations and ultrasound time, and (**F**) ultrasound time and organic phase/aqueous phase ratios on PDI of Gef-Sa-NPs.

Based on these findings, a three-factor, three-level Box–Behnken design (BBD) was employed to further investigate the relationships between the factors and the responses (Table 1) [34]. The particle size and PDI of Gef-Sa-NPs were set as response variables. Using Design Expert 13 software, the relationships between the factors and responses were fitted to a quadratic polynomial model, yielding the corresponding regression equations (Table S5). Three-dimensional response surface plots (Figure 4) were generated to visualize the effects of the various factors on particle size and PDI. The results demonstrated that the organic-to-aqueous phase ratio had a considerable impact on the particle size of Gef-Sa-NPs, whereas the Gef-Sa concentration and sonication time exhibited relatively minor effects.

Using Design Expert 13 software, the formulation was optimized. The optimal preparation conditions for the Gef-Sa-NPs were determined as follows: Gef-Sa concentration of 9 mg/mL, organic-to-aqueous phase ratio of 1:2, and sonication time of 14 min. To evaluate reproducibility, three consecutive batches of Gef-Sa-NPs were prepared according to the optimized process. The results (Table 2) showed that the prepared Gef-Sa-NPs exhibited a uniform particle size of approximately 31 nm, a low PDI of around 0.15, and good batch-to-batch reproducibility, confirming that the optimized process is robust and feasible.

### 3.3. Crystal Structure

**Figure 5:**
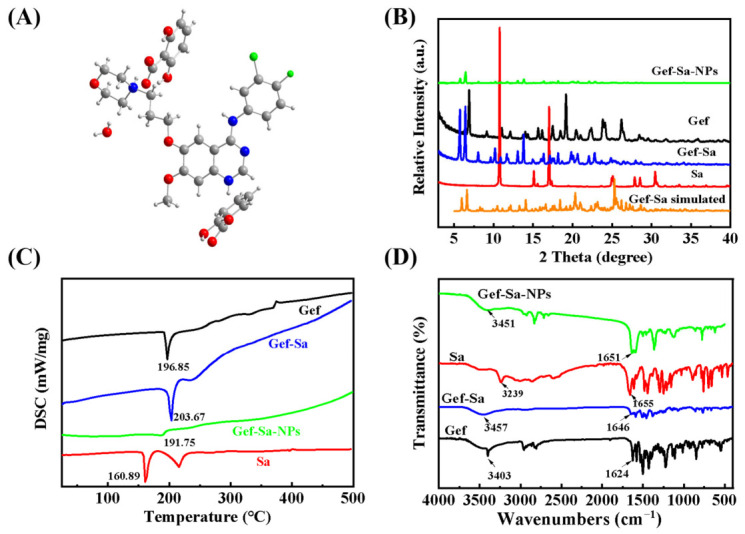
(**A**) Asymmetric unit of Gef-Sa, (**B**) the X-ray diffraction spectra, (**C**) DSC diagrams, and (**D**) FT-IR patterns of Gef, Sa, Gef-Sa and Gef-Sa-NPs.

It can be seen intuitively from Figure 5A that the Gef-Sa crystallizes in the orthorhombic space group *P*2_1_2_1_2_1_. Its asymmetric unit exhibits a Gef:Sa:H_2_O ratio of 1:2:0.5. Notably, the proton from the carboxyl group of Sa transferred to the nitrogen atom on the quinazoline ring of Gef. The primary hydrogen bonds between Gef and Sa molecules include those formed between the nitrogen atom of the morpholine ring and the oxygen atom of the carboxyl group, as well as between the imino group of Gef and the oxygen atom of the carboxyl group. Additionally, hydrogen bonds exist between the hydrogen atoms of the water molecule and the oxygen atoms of the carboxyl group. Meanwhile, an intramolecular hydrogen bond is formed between the hydroxyl hydrogen atom and the carboxyl oxygen atom within the Sa molecule. Consequently, these multiple weak interactions collectively generate a three-dimensional architecture alternately composed of Gef, Sa and solvent water molecules. Crystallographic data and detailed parameters for the Gef-Sa are listed in Table S3, while hydrogen bond angles and distances are provided in Table S4.

### 3.4. PXRD Confirmations

The differences in the PXRD patterns reveal the formation of a new phase. As shown in Figure 5B, comparative analysis of the PXRD patterns of Gef, Sa, Gef-Sa and Gef-Sa-NPs with the computer-simulated pattern of the salt demonstrates that the characteristic peaks of both Gef and Sa disappear in the PXRD pattern of the Gef-Sa. Instead, new diffraction peaks emerge at 2*θ* angles of 5.74°, 6.42°, 8.04°, 9.64°, 10.14°, 11.71°and 13.02°, among others. This phenomenon clearly indicates the formation of a new crystalline phase. The high degree of consistency between the experimental data and the simulated results further confirms the high purity of the Gef-Sa salt. Compared with Gef-Sa, the XRD diffraction peak intensity of Gef-Sa-NPs was weakened, indicating that nanoformulation treatment disrupted the long-range ordered crystal structure of Gef-Sa, resulting in reduced crystallinity [35].

### 3.5. Thermal Behavior Analyses

DSC curves of Gef, Sa, Gef-Sa and Gef-Sa-NPs are illustrated in Figure 5C. Notably, the Gef-Sa exhibits only a single sharp endothermic peak at 203 °C, which is distinct from the melting behaviors of Gef (196.2 °C) and Sa (160.9 °C) [36], indicating the formation of a crystal complex followed by melting decomposition. The appearance of this single endothermic peak confirms the complete transformation of the two components into a novel crystalline phase through crystallization [37]. TG analysis reveals a mass loss of 1.2% for the Gef-Sa salt within the temperature range of 30 °C to 110 °C, corresponding to the presence of half a molecule of water in the system (Figure S2). This finding is consistent with the structure characterized by SCXRD. The melting peak temperature of Gef-Sa-NPs decreased to 191.75 °C, accompanied by peak broadening and reduced thermal effect. This change is attributed to the increased surface energy and decreased crystallinity of the nanoparticles, reflecting the altered thermodynamic characteristics at the nanoscale [38].

### 3.6. FT-IR Spectral Analyses

FT-IR measurement is useful in characterizing specific interactions between components by changes in vibration frequency [39]. Consequently, the FT-IR spectra of Gef, Sa, Gef-Sa and Gef-Sa-NPs are researched to determine the presence of intermolecular hydrogen-bonding interactions. As shown in Figure 5D, the characteristic peak of Gef at 3405 cm^−1^ corresponds to the N-H stretching vibration in the structure, whereas that at 1624 cm^−1^ corresponds to the C=N stretching vibration in the structure; the characteristic peaks of Sa at 3239 cm^−1^ and 1655 cm^−1^ on the FT-IR spectra correspond to the -OH stretching vibration in the structure and the C=O stretching vibration on the carboxyl group, respectively. After the formation of Gef-Sa, the N-H stretching vibration of Gef is shifted to 3457 cm^−1^, while the C=O stretching vibration on the carboxyl group of Sa is shifted to 1646 cm^−1^. It can thus be hypothesized that the N-H of Gef and the C=O of Sa are involved in the formation of the supramolecular synthesizers, which is in agreement with the results of SCXRD. For Gef-Sa-NPs, these peaks further shift to 3451 cm^−1^ and 1651 cm^−1^, respectively, with broadening, confirming hydrogen bonding and van der Waals interactions within the nanoparticles, rather than a simple physical mixture, and these enhanced interactions are critical for nanoformulation formation.

### 3.7. Morphological Analysis of Gef-Sa-NPs

**Figure 6:**
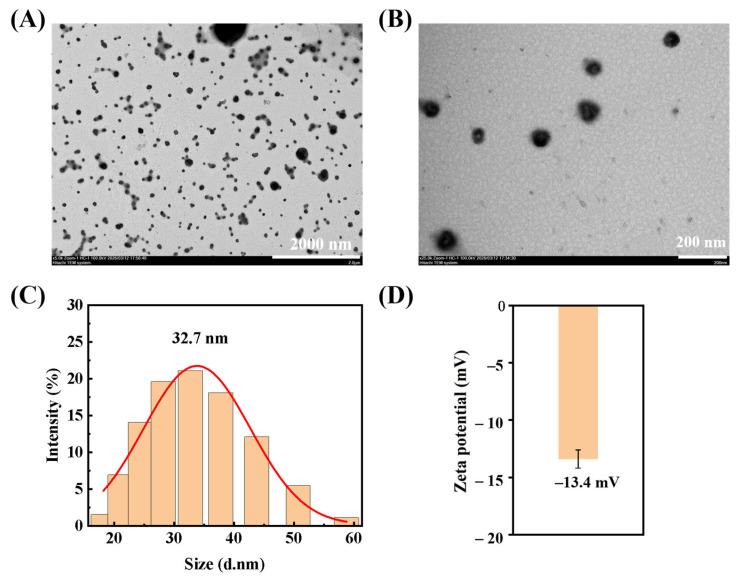
Characterization of Gef-Sa-NPs: (**A**) and (**B**) TEM image; (**C**) particle size distribution; (**D**) Zeta potential.

The micromorphology and particle size distribution of Gef-Sa-NPs were characterized by transmission electron microscopy (TEM) and dynamic light scattering. As presented in Figure 6A,B, Gef-Sa-NPs exhibited a generally spherical or near-spherical morphology with well-defined edges and regular contours, with no distinct core–shell structure or crystalline features observed. The nanoparticles displayed excellent dispersion, with only a small number of dimers or multimers and no large-scale agglomeration, confirming the stable colloidal state of the nanosuspension.

Corresponding to the morphological observations, the particle size distribution curve revealed a narrow and uniform distribution, with a mean particle size of 32.7 ± 1.53 nm with narrow size distribution (PDI = 0.160 ± 0.066) and a zeta potential of −13.40 ± 0.47 mV, indicating good stability (Figure 6C,D) [40]. The size range was predominantly concentrated within 20–50 nm, with only a minor proportion of particles exceeding 50 nm, which could be attributed to slight inter-particle aggregation during sample preparation or measurement. This narrow size distribution aligns with the typical characteristics of well-prepared nanoformulations, and the nanoscale dimensions are expected to facilitate enhanced dissolution and cellular uptake, providing a solid structural basis for subsequent in vitro dissolution and in vivo pharmacodynamic evaluations.

### 3.8. Stability Assessment

**Figure 7:**
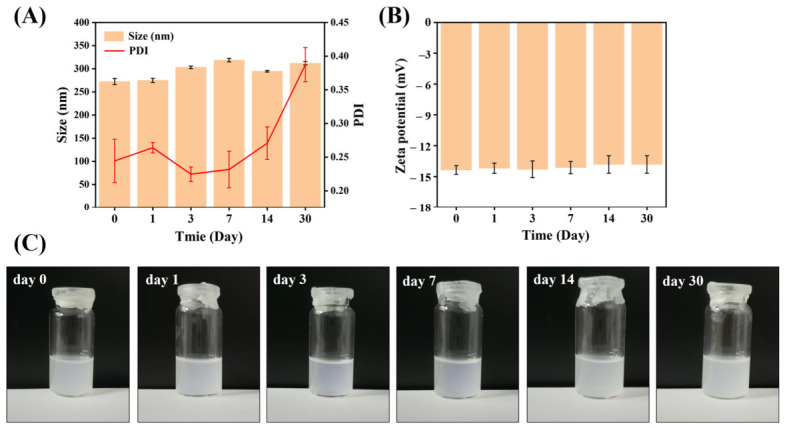
The stability evaluation of Gef-Sa-NPs: (**A**) Particle size and PDI, (**B**) Zeta potential, with representative appearance shown in (**C**). Data are expressed as mean ± SD (*n* = 3).

During the 30-day stability assessment at 4 °C, as presented in Figure 7, the particle size of Gef-Sa-NPs increased sharply from approximately 30 nm to about 270 nm within the first day and then remained relatively stable at around 300 nm for the subsequent 29 days. In contrast, the zeta potential stayed nearly constant at approximately −13 mV throughout the entire period. The initial rapid size increase suggests a fast aggregation process, likely driven by the high surface free energy of the initially small particles (about 30 nm), which promotes irreversible particle–particle interactions in the absence of sufficient steric or electrostatic stabilization. The measured zeta potential of −13 mV is relatively low in absolute value, indicating weak electrostatic repulsion, which fails to prevent particle aggregation under refrigerated conditions. Once the aggregates reached a size of approximately 270–300 nm, no further significant growth was observed, implying that the system reached a metastable state, possibly due to a reduction in the number of free particles and a balance between residual repulsive forces and attractive interactions. The unchanged zeta potential throughout the study indicates that the aggregation was primarily driven by physical interactions (e.g., van der Waals forces or hydrophobic effects) rather than by charge neutralization or surface chemical changes.

### 3.9. In Vitro Drug Release

**Figure 8:**
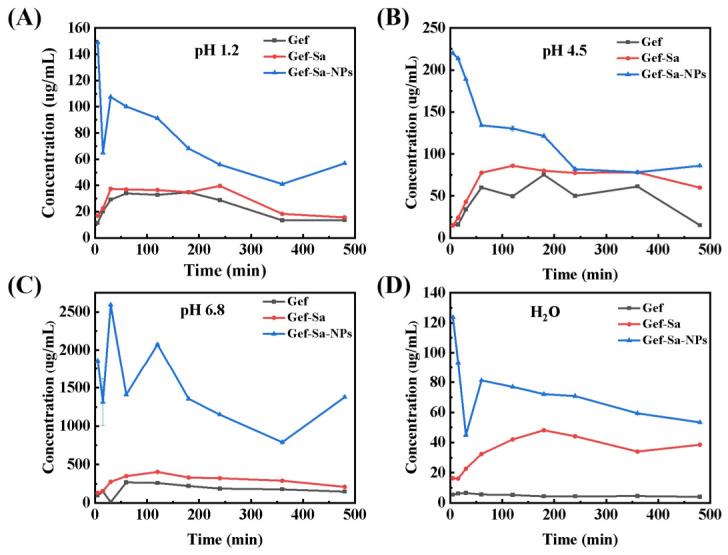
Concentration–time profiles for Gef, Gef-Sa and Gef-Sa-NPs in (**A**) pH 1.2, (**B**) pH 4.5, (**C**) pH 6.8 and (**D**) H_2_O.

This study systematically investigated the dissolution behaviors of Gef, Gef-Sa and Gef-Sa-NPs in pH 1.2, pH 4.5, pH 6.8 and pure water. As depicted in Figure 8, Gef presented the lowest and most gradual dissolution profile in all media, consistent with its inherent poor aqueous solubility and slow dissolution rate as a poorly soluble drug. In contrast, Gef-Sa exhibited markedly improved dissolution rate and concentration compared with Gef, which could be ascribed to the enhanced aqueous solubility and reduced crystallinity achieved via salt formation. Notably, Gef-Sa-NPs demonstrated the most prominent initial dissolution burst in all tested media, reflecting the synergistic advantage of nanonization in amplifying surface area and accelerating dissolution kinetics [41].

All formulations, particularly Gef-Sa-NPs, displayed a characteristic “spring-parachute” dissolution pattern: an initial rapid “spring” phase generated a metastable supersaturated solution within minutes, driven by the high surface energy and large specific surface area of nanoparticles, which led to an abrupt surge in drug concentration. This was followed by a transient decline in concentration, arising from the precipitation of excess drugs as amorphous or microcrystalline species once the equilibrium solubility was exceeded, alongside nanoparticle aggregation and structural rearrangement that reduced the effective dissolving surface area. Subsequently, a sustained “parachute” phase emerged, where the dissolved drug concentration gradually recovered and stabilized over prolonged time, attributed to the slow re-dissolution of the precipitated drug and the sustained release from remaining nanoparticles, as well as the gradual disaggregation of nanoparticle clusters [42].

Notably, the magnitude of the “parachute” effect was highly pH-dependent: in pH 6.8 medium, the recovery of Gef-Sa-NPs concentration was most pronounced, reaching orders of magnitude higher than in other media, owing to the higher intrinsic solubility of the drug under weakly alkaline conditions that facilitated re-dissolution kinetics. In acidic media (pH 1.2, pH 4.5) and pure water, the recovery was relatively moderate due to lower equilibrium solubility and potential accelerated nanoparticle aggregation. These findings collectively demonstrate that the combination of salt formation and nanonization effectively modulates drug supersaturation and crystallization kinetics, thereby significantly enhancing the dissolution performance of Gef, while the observed “spring-parachute” behavior underscores the critical role of supersaturation stabilization in sustaining improved dissolution profiles [43]. Furthermore, the clear trend that faster in vitro dissolution leads to faster in vivo absorption and higher bioavailability provides a foundation for the future establishment of an in vitro–in vivo correlation (IVIVC).

### 3.10. Cytotoxicity Evaluations

To further verify whether the optimized physicochemical properties of the salt form and its nanocrystal form could enhance the antiproliferative activity, the in vitro cytotoxicity of the blank nanoparticles, Sa, Gef, Gef-Sa, Gef+Sa, and Gef-Sa-NPs against A549 cells was investigated using the MTT assay.

**Figure 9:**
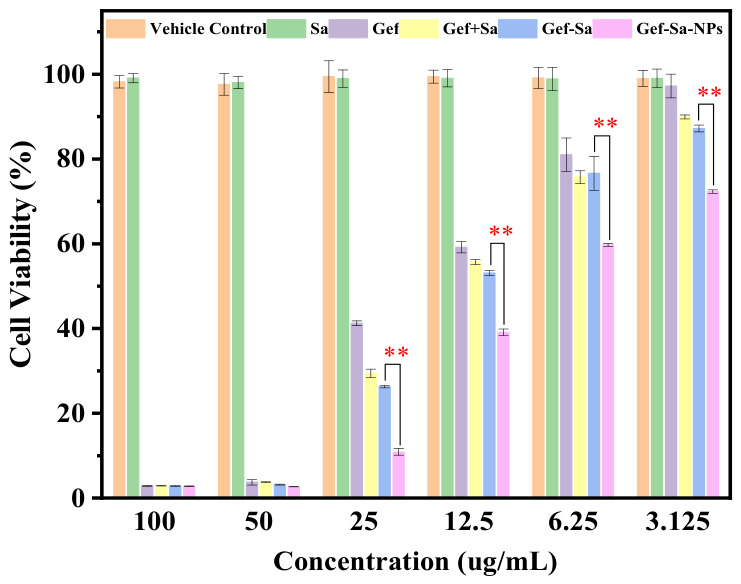
Cell viability of A549 cells treated with vehicle control, Sa, Gef, Gef+Sa, Gef-Sa and Gef-Sa-NPs ranged from 3.125 to 100 ug/mL for 48 h (*n* = 6). * *p* < 0.05, ** *p* < 0.01, *** *p* < 0.001, and **** *p* < 0.0001 relative to the Gef-Sa group.

As shown in Figure 9, at gradient concentrations of 100, 50, 25, 12.5, 6.25, and 3.125 μg/mL, the cell viability in the vehicle control group and the Sa group remained above 95%, indicating that the vehicle and Sa themselves had no obvious cytotoxicity. Compared with the Gef group and the Gef+Sa group, the Gef-Sa and Gef-Sa-NPs groups exhibited significantly greater cell proliferation inhibitory effects at all tested concentrations (*p* < 0.01), and the inhibitory effect weakened with decreasing drug concentration, showing a clear concentration-dependent manner. At high concentrations, all groups showed strong inhibitory effects. When the concentration was reduced to 25 μg/mL, the cell viability in the Gef-Sa-NPs group was only approximately 10%, which was significantly lower than that in the Gef group and the Gef+Sa group. Even at the lowest concentration of 3.125 μg/mL, the Gef-Sa-NPs group still maintained a viability rate of approximately 70%, while the viability in the Gef group was 100%, demonstrating superior long-term antitumor activity.

These results indicate that salt formation significantly enhanced the antitumor activity of Gef, which is closely related to the improvement in drug solubility and cellular uptake efficiency. Furthermore, the nanoformulation strategy further enhanced the cytotoxicity of the salt form, confirming that the biological activity of drugs can be precisely regulated through rational formulation design [44].

### 3.11. Pharmacokinetic Studies of Gef-Sa-NPs

**Figure 10:**
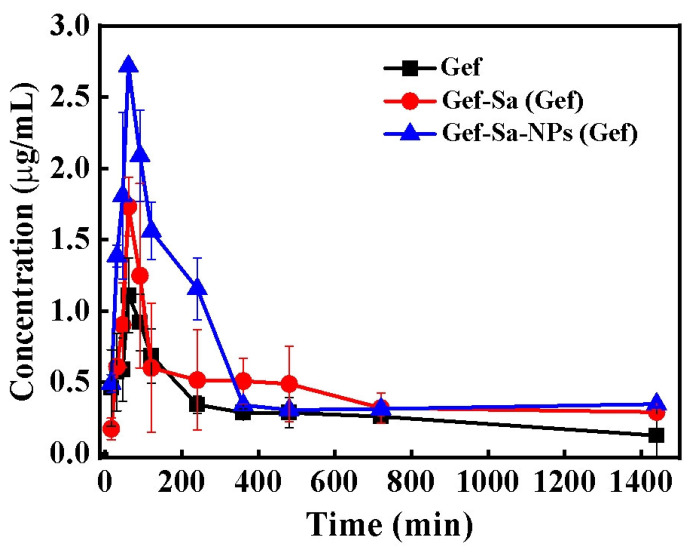
Plasma concentration–time curves of Gef after oral administration of Gef, Gef-Sa and Gef-Sa-NPs. (mean ± SD, *n* = 5).

This study systematically evaluated the effects of different Gef formulations on drug bioavailability through pharmacokinetic experiments in rats, where the concentration of Gef in rat plasma was determined by a validated HPLC method with satisfactory linearity, LLOQ, accuracy, precision, extraction recovery and stability (Figure S1, Table 3, Table 4 and Table 5). The experimental data demonstrated that, compared with the Gef, the Gef-Sa and Gef-Sa-NP formulations significantly increased the systemic drug exposure (Figure 10 and Table 6), with the nanoparticle formulation exhibiting the most favorable pharmacokinetic profile. Specifically, the *AUC*_tot_ of the Gef-Sa reached 614.3 μg/mL·min, representing an approximately 1.5-fold increase relative to the Gef, while the nanoparticle formulation further increased the *AUC*_tot_ to 802.23 μg/mL·min, corresponding to a 1.9-fold improvement in relative bioavailability. This progressive enhancement reveals the significant regulatory effect of formulation strategies on the in vivo behavior of the drug. On one hand, salt formation improves drug solubility by altering the crystal structure; nanonization further enhances the dissolution rate and intestinal absorption efficiency by increasing the specific surface area and modifying surface properties. On the other hand, as gefitinib is a P-gp substrate, the nanoformulation combined with phospholipid stabilizers may indirectly promote drug absorption by inhibiting efflux transport, enhancing mucosal adhesion, and prolonging intestinal retention [45]. Notably, the peak plasma concentration (*C*_max_) of the nanoparticle formulation reached 2.716 ± 0.038 μg/mL, significantly higher than that of the salt formulation (1.732 ± 0.204 μg/mL) and the raw API (1.108 ± 0.261 μg/mL). In addition, the Tmax of Gef-Sa-NPs and Gef-Sa was 30 min, which was shorter than 60 min for Gef, confirming that nanonization and salt formation not only improved the extent of drug absorption but also significantly accelerated the absorption process. The *t*_1/2_ was 60 min for Gef-Sa-NPs, 181 min for Gef, and 456 min for Gef-Sa, respectively. The apparent clearance (CL/F) of Gef-Sa-NPs was lower than those of Gef-Sa and Gef, while the mean residence time (MRT) of Gef-Sa-NPs was significantly shortened, indicating more rapid but sustained absorption and elimination characteristics. All of the pharmacokinetic parameters verified that the salt–nanocrystal dual strategy could effectively optimize the in vivo performance of gefitinib.

## 4. Conclusions

This study innovatively integrates crystal engineering and nanotechnology and successfully develops a novel Gef-Sa multicomponent crystalline nanosuspension system via ultrasound-assisted anti-solvent precipitation, one which exhibits excellent dispersion characteristics. Integrated in vitro dissolution, in vivo pharmacokinetic, and antitumor studies demonstrated that, compared with Gef, the multicomponent crystalline salt significantly enhanced the dissolution rate, bioavailability, and antitumor efficacy. Moreover, nanonization further amplified these properties, highlighting the synergistic effect of formulation design. In summary, building upon the existing crystal structure, this study focuses on the novel construction of nanocrystals and the application of a multi-factor screening approach to systematically address the challenges of the solubility and absorption of Gef. The Gef-Sa-NP combination strategy established in this study provides a research approach for the oral delivery of poorly soluble antitumor drugs. Future research will concentrate on advancing the clinical translation evaluation of this technology and exploring combination therapy regimens to promote its practical application in cancer treatment.

## Figures and Tables

**Table 1:** Factors and levels table of Box–Behnken experimental design.

Factor	Level		
	−1	0	1
A: Gef-Sa concentrations	5	7.5	10
B: Organic phases: aqueous phase	1:2	1:3	1:4
C: Ultrasound times	5	10	15

Note: −1, 0, 1 indicate the low, medium, and high levels, respectively, of each selected factor.

**Table 2:** The reproducibility investigation results of three batches of Gef-Sa-NPs prepared by the optimal prescription.

Batch	1		2		3	
	Size (nm)	PDI	Size (nm)	PDI	Size (nm)	PDI
1	30.30	0.139	31.03	0.139	31.07	0.132
2	35.49	0.235	31.06	0.123	31.68	0.117
3	30.13	0.106	32.84	0.162	31.65	0.114
Mean ± SD	31.97 ± 2.93	0.160 ± 0.066	31.64 ± 1.03	0.141 ± 0.020	31.47 ± 0.34	0.121 ± 0.009
RSD (%)	9.17	41.25	3.26	14.18	1.08	7.44

**Table 3:** Repeatability (intra-day) and intermediate precision (inter-day) of Gef in rat plasma.

QC Level	Nominal Conc. (μg/mL)	Repeatability (Intra-Day, *n* = 6)			Intermediate Precision (Inter-Day, *n* = 6)		
		Mean ± SD (μg/mL)	Precision (RSD, %)	Accuracy (RE, %)	Mean ± SD (μg/mL)	Precision (RSD, %)	Accuracy (RE, %)
Low	1.5	1.002 ± 0.109	10.91	86.12	1.228 ± 0.170	13.84	86.12
Medium	8	7.115 ± 0.767	10.77	87.25	7.165 ± 0.648	9.04	87.25
High	16	14.007 ± 0.944	6.74	87.27	15.575 ± 0.844	5.42	87.27

Note: Data are expressed as mean ± SD. RE, relative error; RSD, relative standard deviation.

**Table 4:** Extraction recovery of Gef from rat plasma (*n* = 5).

QC Level	Nominal Conc. (μg/mL)	Recovery (%)	RSD (%)
Low	1.5	111.7	8.5
Medium	8.0	105.5	6.2
High	16.0	108.7	5.8

**Table 5:** Stability of Gef in rat plasma under various storage conditions (*n* = 3).

Stability Condition	QC Level	Nominal Conc. (μg/mL)	Measured Conc. (μg/mL)	Deviation (%)	RSD (%)
Short-term (RT, 4 h)	Low	1.5	0.979 ± 0.064	+4.23	6.56
	High	16.0	13.121 ± 0.631	−11.23	4.81
Post-preparative (4 °C, 24 h)	Low	1.5	0.971 ± 0.092	+2.09	9.43
	High	16.0	12.855 ± 1.372	−9.97	10.69
Freeze–thaw (3 cycles)	Low	1.5	1.098 ± 0.052	+10.28	4.75
	High	16.0	11.496 ± 1.346	−13.14	11.74

Note: RT, room temperature.

**Table 6:** The pharmacokinetic parameters of Gef in rats after oral administration of Gef, Gef-Sa salt or Gef-Sa-NPs. (mean ± SD, *n* = 5).

Parameters	*C*_max_ (μg/mL)	*T*_max_ (min)	*AUC*_tot_ (μg/mL·min)	*t*_1_/_2_(min)	CL/*F* (mL/min)	*V*z/*F* (mL)	Relative Bioavailability (*F*_rel_)
Gef	1.108 ± 0.261	60	421.79	181	219.7	57,700	/
Gef-Sa	1.732 ± 0.204	60	614.30	456	158.9	104,700	1.5
Gef-Sa-NPs	2.716 ± 0.038 **	60	802.23 **	60 **	117.1 **	10,160 **	1.9

Note: * *p* < 0.05, ** *p* < 0.01, *** *p* < 0.001, and **** *p* < 0.0001 relative to the Gef-Sa group.

## Data Availability

The original contributions presented in this study are included in the article/Supplementary Materials. Further inquiries can be directed to the corresponding authors.

## Supplementary Materials

The following supporting information can be downloaded at https://www.mdpi.com/article/10.3390/pharmaceutics18050572/s1, Figure S1: Standard curve of Gef; Figure S2: TG curve of Gef-Sa; Table S1: Absorbance of Gef in different pH solutions; Table S2: Absorbance of Sa in different pH solutions; Table S3: Crystallographic parameters of Gef-Sa; Table S4: Selected geometric parameters of hydrogen bonds in Gef-Sa; Table S5: Box–Behnken experimental design table and response values. Table S6: Design of Single-Factor Screening Experiments for the Preparation of Gef-Sa-NPs. References [46,47,48,49,50] are cited in Supplementary Materials.

## Abbreviations

The following abbreviations are used in this manuscript:

Gef	Gefitinib
Sa	Salicylic acid
Gef-Sa-NPs	Gefitinib–salicylic acid nanoparticles
TG	Thermogravimetric
DSC	Differential Scanning Calorimetry
SCXRD	Single-crystal X-ray diffraction
PXRD	Powder X-ray diffraction
FT-IR	Fourier transform infrared spectroscopy
NSCLC	Non-small cell lung cancer
EGFR	Epidermal growth factor receptor
API	Active pharmaceutical ingredients
MTT	3-(4,5-Dimethylthiazol-2-yl)-2,5-diphenyltetrazolium bromide
pKa	Dissociation constant
DLS	Dynamic light scattering
TEM	Transmission electron microscopy
LLOQ	Lower limit of quantification
IVIVC	In vitro–in vivo correlation
