# Supplementary material for: Development and In Vitro Evaluation of Gefitinib–Salicylic Acid Nanocrystals for Improved Bioavailability
Source: Pharmaceutics. 2026 May 4;18(5):572. doi: 10.3390/pharmaceutics18050572 (PMC13210458; doi:10.3390/pharmaceutics18050572)
Supplement: Supplementary file 1 [file pharmaceutics-18-00572-s001.zip › pharmaceutics-4248270-supplementary.pdf]

pharmaceutics

Supplementary Information

Development and In Vitro Evaluation of Gefitinib–Salicylic Acid Nanocrystals for Improved Bioavailability

Ling Chen [1,†], Jie-Feng Chen [1,†], Rong Wang [1], Tian-Ran Yang [1], Hao Meng [1], Xin-Xin Zhu [1], Hai-Li Wu [1], Jie-Jie Lai [1], Wei-Wei Chen [1,*], Ning Lin [1,2,*] and Qing Chen [1,2]

[1] College of Pharmacy, Institute of Traditional Chinese and Zhuang-Yao Ethnic Medicine, Guangxi University of Chinese Medicine, Nanning 530200, China; chenling2023@stu.gxtcmu.edu.cn (L.C.); chenjiefeng2024@stu.gxtcmu.edu.cn (J.-F.C.); wangrong2025@stu.gxtcmu.edu.cn (R.W.); yangtianran2025@stu.gxtcmu.edu.cn (T.-R.Y.); menghao2025@stu.gxtcmu.edu.cn (H.M.); zhuxinxin2023@stu.gxtcmu.edu.cn (X.-X.Z.); wuhaili2023@stu.gxtcmu.edu.cn (H.-L.W.); laijiejie2021@stu.gxtcmu.edu.cn (J.-J.L.); chenqing@gxtcmu.edu.cn (Q.C.)
[2] Guangxi Innovation Center of Zhuang Yao Medicine, Nanning 530200, China
* Correspondence: chenww@gxtcmu.edu.cn (W.-W.C.); linning@gxtcmu.edu.cn (N.L.)
† These authors contributed equally to this work.

1. Animal and Ethical Statement

1.1. Experimental Animals and Grouping

This study used fifteen male Sprague-Dawley (SD) rats (specific pathogen-free [SPF] grade, 8 weeks old, body weight 200 - 250 g), purchased from Hunan Slike Jingda Laboratory Animal Co., Ltd. (Laboratory Animal Production License No.: SCXK (Xiang) 2023-0004). All animals were housed under standard conditions of constant temperature (22 ± 2 °C), constant humidity (50 ± 10%), and a 12-hour light/dark cycle, with free access to standard irradiated feed and sterilized drinking water. The rats were randomly divided into 3 groups (n=5): the Gef group, Gef-Sa group, and Gef-Sa-NPs group. All groups received an equivalent dose of gefitinib (Gef, 20 mg·kg^{-1}).

1.2. Ethical Approval and Experimental Conduct

All animal experimental procedures in this study strictly adhered to the relevant regulations of the Chinese Guide for the Care and Use of Laboratory Animals and the U.S. National Institutes of Health Guide for the Care and Use of Laboratory Animals. The experimental protocol was reviewed and approved by the Animal Ethics Committee of Guangxi University of Chinese Medicine (Approval No.: DW20231211-063). The experiments strictly followed the "3Rs" principles (Replacement, Reduction, Refinement) to minimize animal suffering.

1.3. Blood Sample Collection and Processing

All animals were fasted for 12 hours prior to drug administration (with free access to water). Blood samples (approximately 0.5 mL) were collected from the retro-orbital venous plexus at predetermined time points post-administration (15, 30, 45, 60, 90, 120, 240, 480, 720, 1440 min) and placed in heparin sodium anticoagulant tubes. The whole blood samples were centrifuged at 4 °C and 8000 rpm for 10 minutes to separate the plasma. The plasma was then stored at -80 °C until further analysis. Prior to high-performance liquid chromatography (HPLC) analysis, 100 μL of plasma was taken, mixed with 400 μL of methanol for protein precipitation, vortexed thoroughly, and centrifuged at 4 °C and

12000 rpm for 15 minutes. The supernatant was dried under a stream of nitrogen gas, reconstituted in 200 μL of methanol via ultrasonication, and finally, 100 μL of the supernatant was injected for analysis.

2. Drug Concentration Analysis and Data Statistics

2.1. HPLC Analysis Conditions

Chromatographic analysis was performed using an Agilent 1260 Infinity II HPLC system (Agilent Technologies, Santa Clara, CA, USA) equipped with a quaternary pump, autosampler, column thermostat, and UV detector. Separation was achieved on an Agilent C18 column (4.5 mm×250mm，6 μm) maintained at 35°C. The mobile phase consisted of methanol and 0.1% triethylamine in water (75:25, v/v), filtered through a 0.22 μm membrane filter and degassed ultrasonically prior to use. The flow rate was 1.0 mL/min, the detection wavelength was 249 nm, and the injection volume was 10 μL. The total run time for each sample was 10 min, with gefitinib eluting at approximately 6 min. Additionally, the satisfactory linearity, lower limit of quantification (LLOQ), accuracy, precision, extraction recovery, and stability of the analytical method were evaluated.

3. HPLC Method Validation Methods

3.1. Sample Preparation

Stock solution of gefitinib (1 mg/mL) was prepared in methanol and stored at -20°C. Working solutions were prepared by diluting the stock with methanol. Calibration standards were prepared by spiking 100 μL blank rat plasma with 200 μL working solutions to achieve final concentrations of 0.5, 1.0, 5.0, 10.0, and 20.0 μg/mL. QC samples were prepared at low (1.5 μg/mL), medium (8.0 μg/mL), and high (16.0 μg/mL) levels using the same approach.

Aliquots (100μL) of plasma (standards, QC, or study samples) were mixed with 200 μL methanol in 1.5-mL centrifuge tubes, followed by vortex-mixing for 3 min. The mixture was centrifuged at 12,000 rpm for 15 min at 4℃. The supernatant was transferred to clean tubes and evaporated to dryness under a gentle nitrogen stream. The residue was reconstituted in 200 μL methanol with ultrasonication, and 100 μL was subjected to HPLC analysis.

3.2. Method Validation

The analytical method was validated for linearity, lower limit of quantification (LLOQ), repeatability (intra-day precision), intermediate precision (inter-day precision), accuracy, recovery, and stability according to the US Food and Drug Administration (FDA 2024) and National Medical Products Administration (NMPA 2025) guidelines.

3.2.1. Linearity and LLOQ

The calibration curve demonstrated excellent linearity over the concentration range of 0.5-20.0 μg/mL. The typical regression equation was $y = 21.817x + 2.3152$, with a correlation coefficient (r^2) of 0.9999, where y represents the peak area and x represents the gefitinib concentration (μg/mL). The LLOQ was established at 0.5 μg/mL based on the lowest calibration standard with acceptable precision (RSD < 20%) and accuracy (RE within ±20%).

3.2.2. Precision and Accuracy (Repeatability and Intermediate Precision)

The precision and accuracy of the method were evaluated through both intra-day (repeatability) and inter-day (intermediate precision) assessments. Repeatability was assessed by analyzing six replicates (n = 6) of quality control samples at low (1.5 μg/mL), medium (8.0 μg/mL), and high (16.0 μg/mL) concentration levels on the same day under identical analytical conditions. Intermediate precision was determined by analyzing two replicates of each QC level on three consecutive days (n = 6 total), with fresh calibration curves prepared each day to evaluate day-to-day variability. Precision was evaluated using the relative standard deviation (RSD, %), while accuracy was expressed as the relative error (RE, %) comparing the measured concentrations to their nominal values. The acceptance criteria were RSD < 15% and RE within ±15% for both repeatability and intermediate precision according to FDA bioanalytical method validation guidelines.

3.2.3. Recovery

Extraction recovery was determined by comparing peak areas from extracted QC samples (n = 5) with those of reference standards prepared in pure methanol at equivalent concentrations. Recovery values exceeding 100% were attributed to matrix enhancement effects during UV detection and the concentration step during nitrogen evaporation.

3.2.4. Stability

Stability was evaluated under three conditions: (i) short-term stability at room temperature for 4 h; (ii) post-preparative stability at 4°C for 24 h; and (iii) freeze-thaw stability after three complete cycles (−20°C to room temperature). QC samples at low (1.5 μg/mL) and high (16.0 μg/mL) levels were analyzed (n = 3). Samples were considered stable if deviations were within ±15% and RSD < 15%.

4. Pharmacokinetic Analysis

The plasma drug concentration-time curve was plotted with sampling time as the x-axis and plasma Gef concentration as the y-axis. The main pharmacokinetic parameters were calculated using DAS 2.0 pharmacokinetic software.

5. Characterization Methods

5.1. Single-crystal X-ray diffraction (SCXRD)

SCXRD analysis of the Gef-Sa was performed on a Rigaku X-ray single-crystal diffractometer using Cu-Kα radiation (λ = 1.54184 Å). Data collection was carried out using ω-scans and corrected with the CrysAlisPro Program. The structure was solved by direct methods and refined on F2 by full-matrix least-squares methods using the SHELX program package [46, 47].

5.2. Powder X-ray diffraction (PXRD)

PXRD graphs was obtained using a Rigaku MiniFlex 600 diffractometer using Cu-Kα (λ = 1.54186 Å) at 15 mA and 40 kV. The data over the range 3-40°/2θ were collected with a scanning speed of 5o·min^{-1}.

5.3. Thermal Analysis

Differential scanning calorimetry (DSC) and Thermogravimetry (TG) measurements were carried out by NETZSCH STA 449 F5 Simultaneous Thermal Analyzer. The analytical temperature was increased from 26 to 500 oC at a scan rate of 5 oC min−1 under an atmosphere of nitrogen.

5.4. Fourier transform infrared spectroscopy (FT-IR)

FT-IR spectra was detected using a Nicolet Nexus 470 infrared spectrometer (Thermo, USA) with the scanning wavelength set 4000-400 cm-1.

5.5. Particle Characterization of Gef-Sa-NPs

The Z-Ave PDI and zeta potential of the Gef-Sa-NPs were determined using a Zetasizer Nano ZS instrument (Malvern, UK). Data from triplicate measurements are presented as mean ± standard deviation.

5.6. Morphological Analysis of Gef-Sa-NPs

The surface morphology of Gef-Sa-NPs was observed using a Hitachi HT7700 transmission electron microscope (TEM, Tokyo, Japan). The specific sample preparation and observation procedures were as follows: an appropriate amount of the nanosuspension was diluted with ultrapure water and ultrasonically dispersed for 5 min; 10 μL of the dispersion was dropped onto an ultrathin carbon-coated copper grid that had been pretreated with glow discharge, and after adsorption for 2 min, excess liquid was removed with filter paper; after the sample was air-dried, TEM bright-field imaging was performed at an operating voltage of 100 kV.

5.7. Stability Assessment of Gef-Sa-NPs

The stability of Gef-Sa-NPs was assessed over 30 days at 4 °C. Samples were stored sealed and statically under refrigeration. At predetermined time points, aliquots were withdrawn and analyzed for particle size, PDI, and zeta potential.

5.8. In vitro dissolution experiments

The dissolution profiles of Gef, Gef-Sa and Gef-Sa-NPs were evaluated and compared. Suspensions of each sample, containing an equivalent amount of Gef (5mg/mL), were introduced into 25 mL of pH 1.2, pH 4.5, pH 6.8 and pure water [48]. The vessels were maintained at 37 oC with agitation at 100 rpm. At predetermined time intervals (5, 15, 30, 60, 120, 240, 480, 720 and 1440 min), aliquots were withdrawn and immediately filtered through a 0.22 μm microporous membrane. The concentration of Gef in the filtrate was quantified by UV spectrophotometry at a wavelength of 249 nm [49].

5.9. Anti-tumor activity assays

The human non-small cell lung cancer cell line A549 was cultured in DMEM medium containing FBS, penicillin and streptomycin in a humidified atmosphere of 5 % CO_2 at 37 oC. The tumor cell strains in the logarithmic growth phase were seeded into a 96-well plate for 24 h. The medium was then replaced with fresh medium containing blank nanoparticles (Vehicle Control), Sa, Gef, Gef-Sa, Gef+Sa, and Gef-Sa-NPs at the different concentrations. After 48 h of drug exposure, 10 μL of 5 mg mL^{-1} MTT solution was added to each well and incubation continued for 4 h. The formazan crystals were dissolved with 150 μL DMSO, and absorbance was read at 490 nm. Cell viability was calculated as:

Cell viability (%) = [(As −Ab) / (Ac −Ab)] ×100%

As: Absorbance of wells with cells, MTT solution, and drug treatments.

Ab: Absorbance of wells with medium and MTT solution without cells.

Ac: Absorbance of wells with cells, MTT solution without drug treatments.

5.10. In vivo pharmacokinetic assays

The in vivo experiment in rats was strictly conducted in accordance with the Regulations for the Administration of Experimental Animals and was supervised by the Experimental Animal Center of Guangxi University of Chinese Medicine. Fifteen male SD rats (weighing 200-250 g) were fasted for 12 hours before the experiment (with free access to water) and used to evaluate pharmacokinetic parameters, and each group was administered Gef, Gef-Sa, or Gef-Sa-NPs by oral gavage at a dose of 20 $mg{\cdot}kg^{-1}$ (calculated as gefitinib).

After administration, approximately 0.5 mL of blood was collected from the orbital sinus of rats at nine time points: 15, 30, 45, 60, 90, 120, 240, 480, 720, and 1440 min. The blood samples were placed in heparinized tubes, immediately stored at 4 °C, and then centrifuged at 8000 r/min for 10 min. The upper plasma layer was separated, transferred to clean centrifuge tubes, and stored at -20 °C until analysis.

Before analysis, the plasma samples were pretreated as follows: 100 μL of plasma was mixed with 200 μL of methanol, vortexed for 1 min to precipitate proteins, and centrifuged at 12,000 r/min for 15 min. The supernatant was collected, filtered through a 0.22 μm membrane, and the drug concentration was determined by high-performance liquid chromatography (HPLC) [50].

5.11. Statistical analysis

All data were presented as the means standard deviation (SD) and the statistical analysis were assessed using GraphPad Prism 9.0 to calculate significant difference between different groups. The Student's t-test was utilized to compare the means of two groups, whereas the one-way ANOVA was assessed in comparing more than two groups. $P < 0.05$ was considered a significant difference.

6. Result

Table S1. Absorbance of Gef in different pH solutions.

Number	pH	Absorbance 218 nm
1	1.53	0.49137
2	2.20	0.48694
3	3.13	0.48215
4	4.27	0.47164
5	5.30	0.46889
6	6.13	0.45197
7	6.79	0.43831
8	7.27	0.40289
9	8.17	0.36593
10	9.18	0.35462
11	10.36	0.34519

Table S2. Absorbance of Sa in different pH solutions.

Number	pH	Absorbance 236 nm
1	1.21	0.70005
2	1.53	0.65516
3	2.2	0.64588

4	3.13	0.53043
5	4.27	0.49319
6	5.3	0.49123
7	6.13	0.49097
8	6.79	0.48949
9	7.27	0.4855
10	8.17	0.47561
11	9.18	0.47465

Table S3. Crystallographic Parameters of Gef-Sa.

**Compound**	**Gef-Sa**
Empirical formula	$C_{72}H_{72}Cl_2F_2N_8O_{19}$
Formula weight	1462.27
Crystal system	Orthorhombic
Space group	$P2_12_12_1$
a/Å	14.34500(10)
b/Å	17.79890(10)
c/Å	26.66400(10)
α/°	90
β/°	90
γ/°	90
Volume/Å^3	6807.99(7)
Z	4
ρ_{calc}g/cm^3	1.427
μ/mm^{-1}	1.596
Crystal size/mm^3	0.2×0.1×0.05
Reflections collected	18623
Independent reflections	11299 [R_{int} = 0.0130, R_{sigma} = 0.0196]
Data/restraints/parameters	11299/29/957
Goodness-of-fit on F^2	1.036
Final R indexes [$I > 2\sigma(I)$]	R_1 = 0.0538, wR_2 = 0.1505
Final R indexes [all data]	R_1 = 0.0549, wR_2 = 0.1520
Largest diff. peak/ hole / e Å^{-3}	0.91/-0.39

$R_1 = F_o - F_c/F_o$. $wR_2 = [w(F_o^2 - F_c^2)^2/w(F_o^2)^2]^{1/2}$

Table S4. Selected Geometric Parameters of Hydrogen Bonds in Gef-Sa.

**D-H···A**	**d(D···H)/Å**	**d(H···A)/Å**	**d(D···A)/Å**	**D-H···A/°**
N1- H1···O7^1	0.86	2.01	2.839(4)	161.5
N3– H3···O14	0.86	1.82	2.678(4)	173.9
N4– H4···O17	0.98	1.71	2.664(5)	163.8
N5– H5A···O8	0.86	1.99	2.806(5)	158.7
N7– H7···O10	0.86	2.37	2.964(6)	126.9
N7– H7···O11	0.86	1.93	2.783(6)	171.2
N8– H8···O13	0.98	1.78	2.756(5)	173.9
N8– H8···O14	0.98	2.47	3.133(5)	124.7
O1W–H1WA···O11	0.85	2.04	2.859(8)	160.7
O1W–H1WB···O16^2	0.85	2.14	2.941(9)	158.1
O9– H9···O7	0.82	1.83	2.554(5)	146.8
O12–H12···O10	0.82	1.77	2.490(7)	145.3

O15–H15···O13	0.82	1.90	2.619(5)	145.1
O18–H18···O16	0.82	1.90	2.627(10)	146.4
O19–H19···O17	0.82	1.81	2.521(9)	144.6

Symmetry code: [1] -1/2+*X*, 3/2-*Y*, 1-*Z*; [2]1/2+*X*, 1/2-*Y*, 1-*Z*

Table S5. Box-Behnken experimental design table and response values.

Number of times	Factor			Response value	
	A	B	C	Size	PDI
1	7.5	2	5	40.68	0.299
2	10	3	5	58.00	0.422
3	5	3	5	75.73	0.470
4	7.5	4	5	68.06	0.300
5	10	2	10	33.93	0.196
6	5	2	10	48.27	0.403
7	7.5	3	10	46.46	0.400
8	7.5	3	10	61.73	0.483
9	7.5	3	10	60.30	0.446
10	7.5	3	10	51.93	0.421
11	7.5	3	10	54.19	0.436
12	10	4	10	66.53	0.467
13	5	4	10	72.75	0.443
14	7.5	2	15	30.54	0.091
15	10	3	15	37.18	0.325
16	5	3	15	59.72	0.496
17	7.5	4	15	49.83	0.390

Size: Y=54.92-7.6A+12.97B-8.15C+2.03AB-1.20AC-2.02BC+5.41A^2-4.97B^2 -2.68C^2

(model: F=8.97, P=0.0043, P<0.05, significant; lack-of-fit: F=0.6059, P=0.6453, P>0.05, not significant).

PDI: Y=0.4372-0.0502A+0.0764B-0.0236C+0.0577AB-0.0308AC+0.0745BC+0.0492A^2-0.1091B^2-0.0581C^2

(model: F=33.60, P<0.0001, significant; lack-of-fit: F=0.1581, P=0.9192, P>0.05, not significant).

Table S6. Design of Single-Factor Screening Experiments for the Preparation of Gef-Sa-NPs.

Trial	Gef-Sa concentrations (mg/mL)	Concentrations of stabilizer (%)	Organic phases: aqueous phase	Ultrasonic powers (%)	Ultrasound times (min)	Ultrasonic probe depths (cm)
1	1	0.5	1:5	20	10	0.5
2	2.5	0.5	1:5	20	10	0.5
3	5	0.5	1:5	20	10	0.5
4	7.5	0.5	1:5	20	10	0.5
5	10	0.5	1:5	20	10	0.5
6	7.5	0.1	1:5	20	10	0.5
7	7.5	0.25	1:5	20	10	0.5
8	7.5	0.5	1:5	20	10	0.5
9	7.5	0.75	1:5	20	10	0.5
10	7.5	1	1:5	20	10	0.5
11	7.5	0.75	1:1	20	10	0.5
12	7.5	0.75	1:2	20	10	0.5
13	7.5	0.75	1:3	20	10	0.5

14	7.5	0.75	1:4	20	10	0.5
15	7.5	0.75	1:5	20	10	0.5
16	7.5	0.75	1:2	10	10	0.5
17	7.5	0.75	1:2	15	10	0.5
18	7.5	0.75	1:2	20	10	0.5
19	7.5	0.75	1:2	25	10	0.5
20	7.5	0.75	1:2	30	10	0.5
21	7.5	0.75	1:2	25	10	0.5
22	7.5	0.75	1:2	25	15	0.5
23	7.5	0.75	1:2	25	20	0.5
24	7.5	0.75	1:2	25	25	0.5
25	7.5	0.75	1:2	25	30	0.5
26	7.5	0.75	1:2	25	15	0
27	7.5	0.75	1:2	25	15	0.5
28	7.5	0.75	1:2	25	15	1

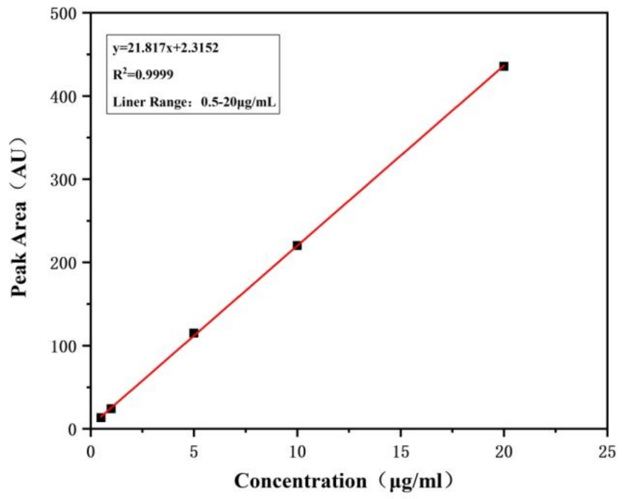

Figure S1. Standard curve of Gef.

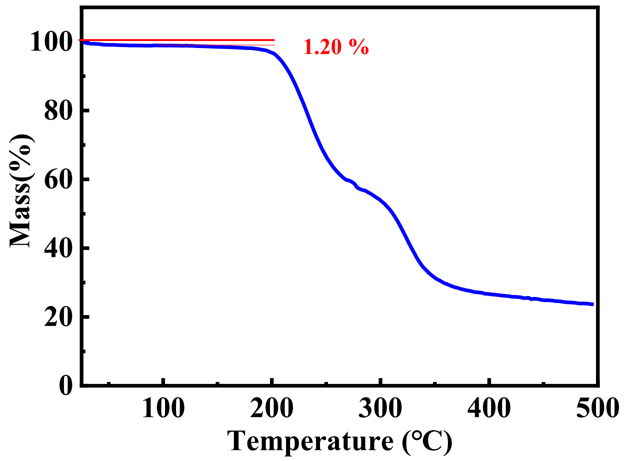

Figure S2. TG curve of Gef-Sa.
